# Supplementary material for: Visualization of Nuclease- and Serum-Mediated Chromatin Degradation with DNA–Histone Mesostructures
Source: Int J Mol Sci. 2023 Feb 6;24(4):3222. doi: 10.3390/ijms24043222 (PMC9959986; doi:10.3390/ijms24043222)
Supplement: Supplementary file 1 [file ijms-24-03222-s001.zip › ijms-2123471-supplementary.pdf]

Supplementary Materials

Visualization of nuclease-mediated chromatin degradation with DNA-histone mesostructures

Supplementary Methods

Fluorescent imaging of NETs with SYTOX Green

$1x10^5$ normal control neutrophils in serum-free RPMI media supplemented with L-glutamine (Gibco; Thermo Fisher Scientific, Waltham, MA, USA) were seeded onto 0.001% poly-L-lysine coated (P4707; Sigma-Aldrich, St. Louis, MO, USA) coverslips. NET formation was induced by incubating neutrophils with 20 nM PMA (Sigma-Aldrich, St. Louis, MO, USA) for 4 hours at 37°C and 5% CO_2. Following stimulation, culture media was gently aspirated, and cells were washed with 1x phosphate-buffered saline (PBS). For fluorescent staining, cells were incubated with 1 uM SYTOX Green (S7020; Thermo Fisher Scientific, Waltham, MA, USA) for 30 minutes at 4°C and washed once with 1x PBS. Cells were counterstained with nuclear DNA staining with Hoechst 33342 (H3570, Thermo Fisher Scientific, Waltham, MA, USA) for 10 minutes at room temperature. Hoechst was replaced with fresh 1x PBS and images were collected with a Cytation 5 Cell Imaging Multi-Mode Reader (BioTek, Santa Clara, CA, USA).

Degradation by nuclease buffers, EDTA and BSA

The supplemental procedures used in this section follow this article's DHM fabrication and degradation assay procedures, with minor reagent modifications. Sample preparation. DHMs were incubated with nuclease buffer composed of 10 mM Tris-HCl, 10 mM $MgCl_2$, 2 mM $CaCl_2$, and 150 mM NaCl at a pH of 7.5. To better understand the nuclease activity of serum samples, the nuclease buffer was compared with a magnesium and calcium-free nuclease buffer with the following composition: 10 mM Tris-HCl, and 150 mM NaCl at a pH of 7.5. Healthy control serum samples were diluted to 5% in both buffers for DHM degradation treatments. For chelating experiments, DHMs were treated with 50 mM EDTA (D4263; Sigma-Aldrich, St. Louis, MO, USA). To observe the effect of BSA on DHM structure, BSA was diluted to 1.25, 3%, or 5% BSA (126609; EMD Millipore, Burlington, MA, USA) in nuclease buffer. Additionally, for comparison, healthy control serum was diluted to 5% in each of these buffers.

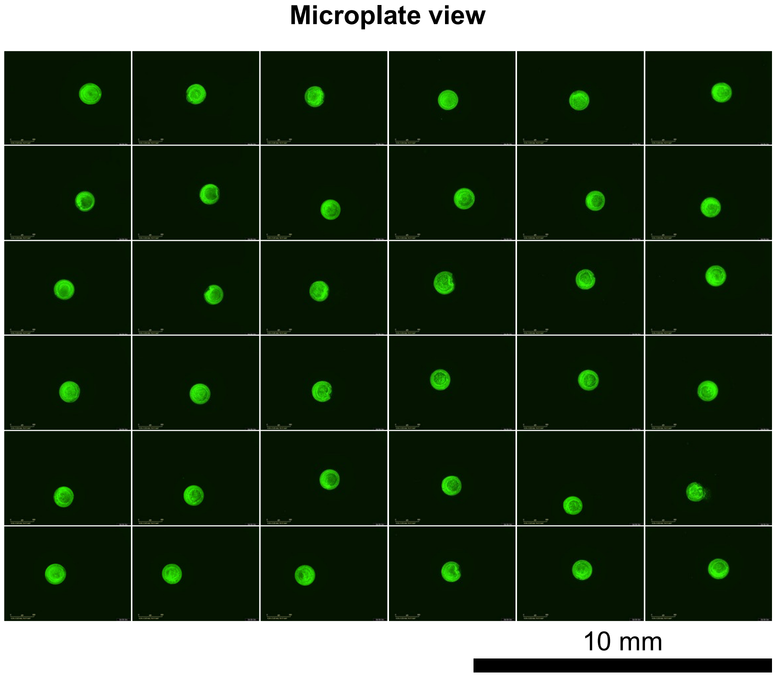

Supplementary Figure S1. DNA-histone mesostructures (DHMs) patterned into each well of a 96-well plate. Each DHM structure was rehydrated and stained with SYTOX Green and washed. Images representing the initial state of the degradation assay were captured with the instrument Incucyte® S3 in the green fluorescent channel with a 4X objective. The scale bar is 10 mm.

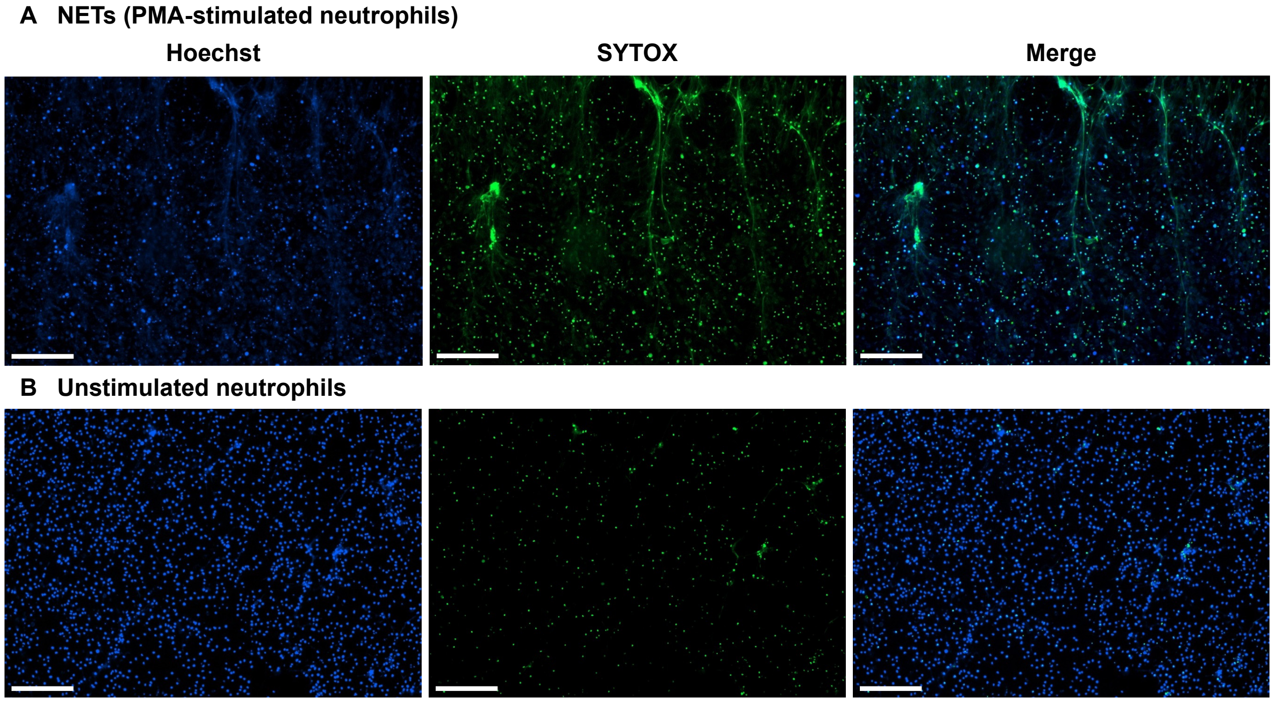

Supplementary Figure S2. Visualization of cell-derived neutrophil extracellular traps (NETs) on coverslips stained with SYTOX Green. **(A)** Neutrophils, labeled with Hoechst 33342 in blue, were stimulated with 20 nM phorbol 12-myristate 13-acetate (PMA), leading to cellular decondensation and fibrous NETs that are visualized with SYTOX Green. **(B)** Unstimulated neutrophils did not form NETs. The scale bar is 300 μm.

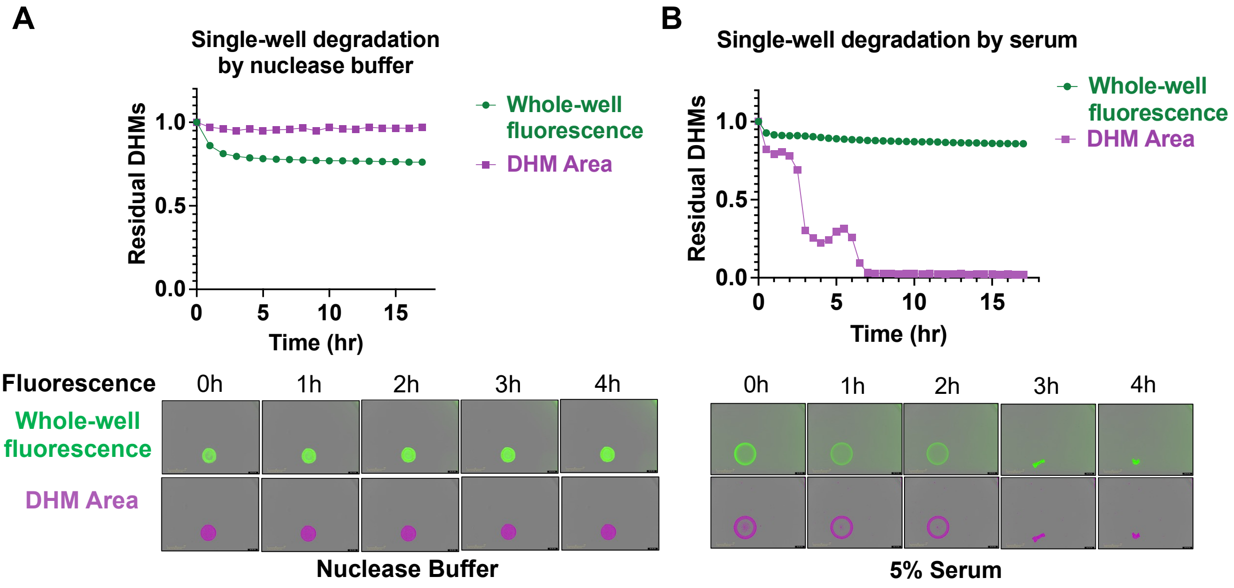

Supplementary Figure S3. Object area analysis depicts DHM structure degradation by additional conditions. The DHM degradation kinetics of a single structure incubated with nuclease buffer background treatment **(A)** and 5% normal human serum **(B).** The kinetic curve of the whole-well fluorescence, in green, is compared with the DHM area, in magenta. The DHM area depicts the object area mask by image segmentation analysis. The corresponding images of plotted points are displayed as a time course (bottom panels). The collected green fluorescent images have a high nonspecific signal (top panel, normalized GFU values in white). Therefore, Incucyte® Software image analysis was applied to segment the object area in each image (magenta traces, bottom panel, normalized area values in white). The scale bar is 800 μm.

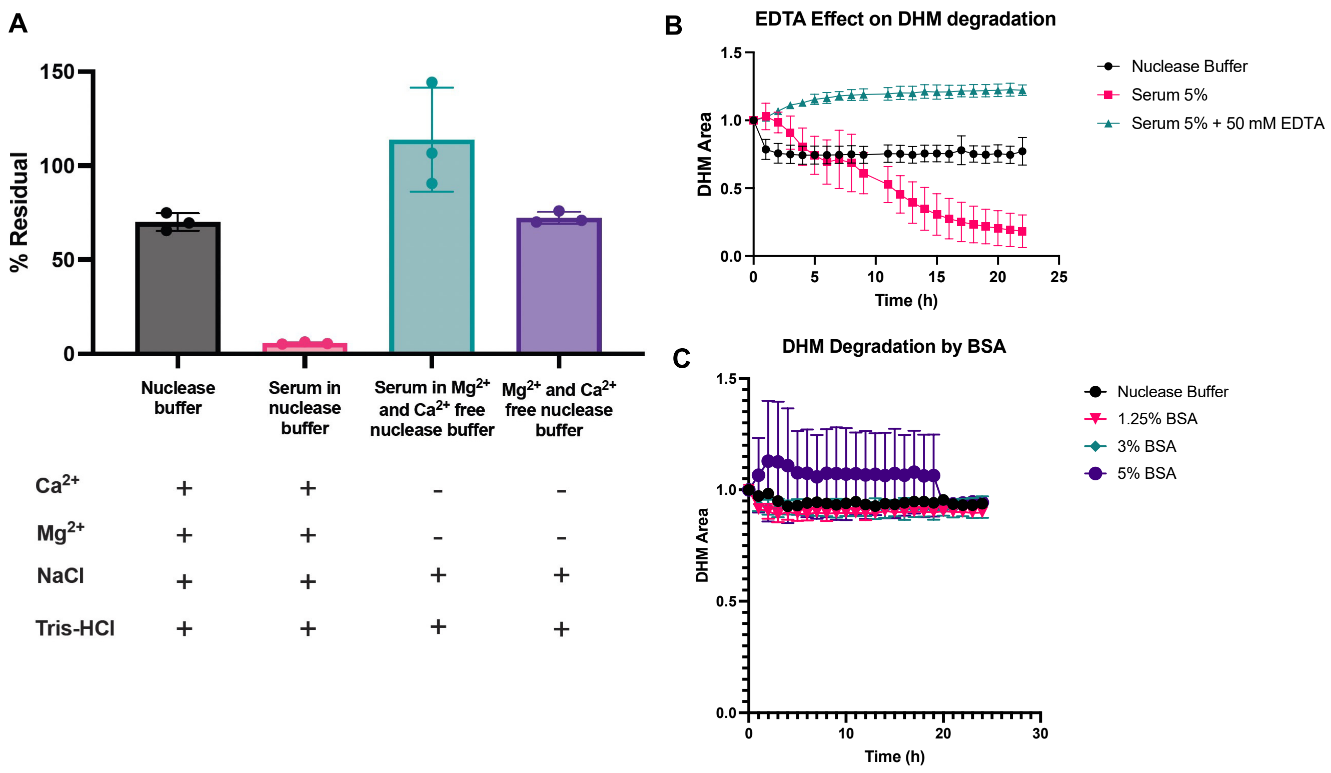

Supplementary Figure S4. DHM degradation by nuclease buffers, EDTA, and bovine serum albumin (BSA) solutions. **(A)** Serum degradation of DHMs is dependent on calcium and magnesium cations. DHMs were not degraded by nuclease buffers within the 24-hour incubation. DHMs were degraded by 5% serum only in the nuclease buffer containing magnesium and calcium ions. Each point is representative of n=3 samples. **(B)** DHM degradation by 5% serum was inhibited upon co-incubation with 50 mM EDTA. Each plotted point is representative of n=6 samples per treatment. **(C)** DHM degradation by BSA diluted in nuclease buffer. The tested concentrations of 1.25%, 3%, and 5% BSA did not degrade DHMs. Each point is representative of n=2 samples.
